# Effects of positive end-expiratory pressure and oxygen concentration on non-hypoxemic apnea time during face mask ventilation of anesthesia induction: A randomized controlled trial

**DOI:** 10.3389/fphys.2022.1090612

**Published:** 2023-01-09

**Authors:** Chunxiang Hao, Xiaojing Ma, Xiangmei Piao, Yunke Fu, Libin Ma, Weidong Mi, Lorenzo Berra, Changtian Li, Changsheng Zhang

**Affiliations:** ^1^ Department of Anesthesiology, First Medical Centre of Chinese PLA General Hospital, Beijing, China; ^2^ Department of Anesthesia, Critical Care and Pain Medicine, Massachusetts General Hospital, Boston, MA, United States; ^3^ Faculty of Hepato-Pancreato-Biliary Surgery, Chinese PLA General Hospital, Beijing, China

**Keywords:** positive end-expiratory pressure (PEEP), fraction of inspiration O_2_ (FiO_2_), preoxygenation, non-hypoxemic apnea time, mechanical ventilalion

## Abstract

**Background:** The optimal ventilatory strategy for the face mask ventilation during anesthesia induction is still unknow.

**Methods:** We evaluated the effect of two positive end-expiratory pressure (PEEP) levels (0 cmH_2_O and 6 cmH_2_O) and two oxygen concentration levels (1.0 and .6) on non-hypoxemic apnea time during face mask ventilation of anesthesia induction. Sixty adult patients scheduled for elective surgery were enrolled in this study. The patients were randomized to receive anesthesia induction with four different ventilation strategy under volume-controlled ventilation. Patients assigned to the LOZP group received low fraction of inspiration O_2_ (FiO_2_ = .6) and 0 PEEP. Patients assigned to the LOHP group received low fraction of inspiration O_2_ (FiO_2_ = .6) and 6 cmH_2_O PEEP. Patients assigned to the HOZP group received high fraction of inspiration O_2_ (FiO_2_ = 1.0) and 0 PEEP. Patients assigned to the HOHP group received high fraction of inspiration O_2_ (FiO_2_ = 1.0) and 6cmH_2_O PEEP. After 3 min of ventilation, the patient was intubated but disconnected from the breathing circuit. Ventilation was not initiated until the pulse oximetry dropped to 90%. The primary outcome was non-hypoxemic apnea time defined as the time from cessation of ventilation to a pulse oximeter reading of 90%. The secondary outcome was the PaO_2_/FiO_2_ ratio immediately after ventilation.

**Results:** The non-hypoxemic apnea time was significantly longer in the group of HOHP when compared to the other three groups (192 s ± 70 s, 221 s ± 74 s, 284 s ± 101 s, and 353 s ± 85 s in the LOZP, LOHP, HOZP, and HOHP group, respectively). The PaO_2_/FiO_2_ ratio immediately after ventilation was significantly higher in the group of LOHP when compared to the other three groups (LOZP 393 ± 130, LOHP 496 ± 97, HOZP 335 ± 58, HOHP 391 ± 50). When compared the PaO_2_/FiO_2_ ratio immediately after ventilation to its value before administration of anesthesia, the PaO_2_/FiO_2_ ratio in the group of LOHP was improved, the group LOZP and HOHP remained the same, while the group HOZP significantly decreased.

**Conclusion:** Application of PEEP and 100% of oxygen during face mask ventilation of induction could maximize the non-hypoxemic apnea time. However, the use of PEEP and 60% of oxygen during preoxygenation resulted in improved PaO_2_/FiO_2_ ratio.

## 1 Introduction

Induction of anesthesia is of crucial importance to both patient and anesthesiologists. One of the biggest challenges to be deal with during this period is endotracheal intubation, especially in patients with difficult airway or significant lung comorbidities. Hypoxemia may occur due to multiple attempts of endotracheal intubation, low oxygen reserve capacity or poor pulmonary gas exchange (Merry et al., 2009). Therefore, prolongation of non-hypoxemic apnea time and improvement of arterial partial pressure of oxygen is important to provide safe anesthetic induction.

At present, the standard face mask ventilation strategy after preoxygenation and induction of anesthesia is to apply bag-squeezing technique with 100% oxygen, which had been used for a long time since the establishment of modern general anesthesia. However, the combination of high oxygen fraction (FiO_2_), muscle relaxant and positive pressure ventilation accelerate the formation of atelectasis in dependent lung regions and reduction of end-expiratory lung volume during induction period, which further hindered the ventilation efficiency of the patient (Hedenstierna et al., 2019). Recent studies showed that applying PEEP during anesthesia induction alleviate formation of lung atelectasis, and effectively prolongs apnea time (Kim et al., 2021). It is also reported that application of low oxygen fraction could reduce atelectasis and improve PaO_2_/FiO_2_ perioperatively (Koo et al., 2019). However, to date there are no studies on effects of both PEEP and oxygen concentration on non-hypoxemic apnea time and PaO_2_/FiO_2_ ratio during face mask ventilation of anesthetic induction.

Therefore, in this study, we aimed to assess the effect of two positive PEEP levels (0 cmH_2_O and 6 cmH_2_O) and two FiO_2_ levels (1.0 and .6) on non-hypoxemic apnea time and PaO_2_/FiO_2_ ratio during face mask ventilation of anesthesia induction in adults.

## 2 Methods

This was a prospective, randomized controlled trial performed at Anesthesia and Operation Centre, First Medical Centre of Chinese PLA General Hospital. This study was approved by the Ethical Committee of Chinese PLA General Hospital in Beijing, China (protocol number: S2021-489-01, president of the ethics committee: Prof. Xiangyang Chu, Date of approval: 30 Sep 2021). Written informed consent was obtained from all subjects participating in the trial. The trial was registered prior to patient enrolment at the Chinese Clinical Trial Registry (ChiCTR2100053037, Principal investigator: Changsheng Zhang, Date of registration: 10 Nov 2021). This manuscript adheres to the applicable CONSORT guidelines.

### 2.1 Study population

From 11 Nov 2021 to 30 Dec 2021, 60 patients aged 18–80 years were enrolled in this study. We included patients who were scheduled for surgery under general anesthesia with endotracheal intubation. Further inclusion criteria were: American Society of Anesthesiology (ASA) Class I or II, age 18–80 years, and a body mass index less than 35 kg/m^2^. Patients were excluded if they had one or more of the following criteria: indwelling nasal gastric tube, anticipated difficult intubation, obstructive sleep apnea, history of documented chronic organ failure, hypertension, ischemic heart disease, atrioventricular block, incomplete or partial heart blocks.

### 2.2 Study procedures

All the anesthesiologists and investigators participating in this study had at least five years of experience as attending physicians at our institution. Eligible patients will be randomly assigned to four groups prior to their electively scheduled surgery with an allocation ratio of 1:1:1:1. Restricted randomization assignments was determined by a computerized random-number generator. The patient and investigator in the operating room were blinded to the patient allocation and intervention until all trial data were analyzed.

After arriving in the operation room, the patient was placed supine on the induction table. Venous access was obtained with an 18-gauge cannula placed in the left forearm. Electrocardiogram, pulse oximetry, and non-invasive blood pressure (cuff placed on the right upper arm) were continuously monitored. Under strict aseptic precautions, the radial artery was then cannulated with a 22-gauge cannula after local infiltration with 2% lidocaine and the arterial line flushed with heparinized saline. Baseline values of heart rate, blood pressure and SpO_2_ were recorded, and a sample of arterial blood gas (ABG1) was taken with the patient breathing room air.

Before anesthesia induction, patients were instructed to take five deep breaths under room air, while the whole breathing circuit (including the face mask) was filled with oxygen. Then, anesthesia was induced by anesthesiologist in charge of the case using midazolam .03 mg/kg, sufentanil .25 μg/kg, propofol 2 mg/kg, rocuronium .6 mg/kg. Ventilation was initiated after patients were unresponsive *via* an appropriately sized face mask, provided by an anesthesia machine (Fabius plus, Drager, AG, Lubeck/Germany). A “Guedel” style oropharyngeal airway was placed to ensure sufficient tidal volume. Ventilation was considered to be successful when the end-tidal oxygen concentration (E_t_O_2_) achieved 90% of the setting FiO_2_ and the patient’s expired tidal volume was similar to the volume set on the ventilator. To avoid gastric insufflation and aspiration, Sellick maneuver was applied during ventilation by an anesthesia nurse.

All the patients in this study received volume-controlled ventilation during ventilation for 90 s with a tidal volume of 6 mL/kg (ideal body weight), and a respiratory rate of 16 bpm–20 bpm, inspiration/expiration ratio of 1:2 and a gas flow rate of 10 L/min. Patients assigned to the LOZP group (Low FiO_2_ + ZEEP) received FiO_2_ = .6 and 0 PEEP. Patients assigned to the LOHP group (Low FiO_2_ + PEEP) received FiO_2_ = .6 and 6 cmH_2_O PEEP. Patients assigned to the HOZP group (High FiO_2_ + ZEEP) received FiO_2_ = 1.0 and 0 PEEP. Patients assigned to the HOHP group (High FiO_2_ + PEEP) received FiO_2_ = 1.0 and 6 cmH_2_O PEEP. A sample of arterial blood was obtained at the end of the 90 s mechanical ventilation period (ABG2).

At the end of the allocated mechanical ventilation for 90 s, the patient was intubated with an appropriately sized endotracheal tube using a video laryngoscope (VL310, UE Medical Corp., Zhejiang, China) but was not ventilated until pulse oximetry saturation reached 90%. The time from cessation of ventilation to a pulse oximeter reading of 96% (T_100-96_) and 96%–90% (T_96-90_) was recorded, respectively. When the pulse oximetry saturation reached 90%, mechanical ventilation was resumed with a tidal volume of 6 mL/kg, 12 breaths per minute, FiO_2_ = .6, 6 cmH_2_O PEEP and a gas flow rate of 2 L/min. The lowest pulse oximeter reading was recorded during this period. The time from resume of mechanical ventilation to a pulse oximeter reading of 96% (T_min-96_) and 96%–100% (T_96-100_) was also recorded. If the patient took more than 120s from a pulse oximeter reading of 96%–100%, a recruiting breath was applied.

The primary outcome was non-hypoxemic apnea time defined as the time from cessation of ventilation to a pulse oximeter reading of 90% (T_100-90_). The secondary outcome was the PaO_2_/FiO_2_ ratio at the time when ventilation was discontinued just prior to intubation. This study was a double-blind randomized controlled study where the patient and outcome assessor were blinded to study interventions until the study was completed.

### 2.3 Statistical analysis

According to our pilot study including 12 patients, the non-hypoxemic apnea time in the group LOZP, LOHP, HOZP, and HOHP was 190 s, 250 s, 248 s, and 336 s, respectively (standard deviation 99.8 s). Sample size calculations showed that 12 patients were needed in each group (a power of .8 and a two-sided *p*-value of less than .05). Therefore, we anticipated enrolling 15 subjects (12 + 20% possible dropouts) in each group.

The statistical analysis was conducted using Statistical Package for Social Sciences (SPSS Inc., Chicago, IL, Version 17.0 for Windows). Results are expressed as means and standard deviations, medians and ranges, or numbers and percentages. Repeated measures analyses of variance (ANOVAs) were used to analyze the difference of non-hypoxemic apnea time and PaO_2_/FiO_2_ ratio between each group. Other results were subjected to one-way ANOVA using the Tukey multiple comparison test. Normality of data was checked by measures of skewness and Kolmogorov Smirnov tests. A *p*-value of less than .05 was considered statistically significant.

## 3 Results

**FIGURE 1:**
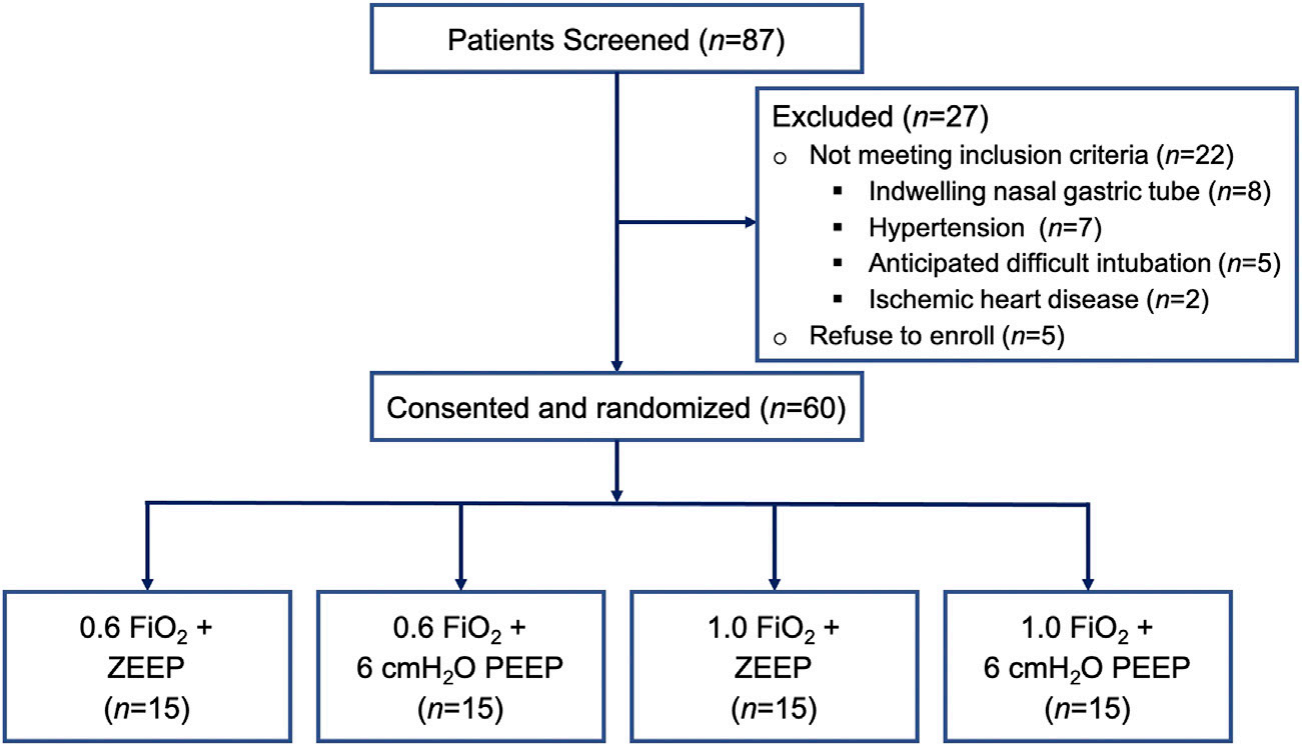
Flow chart of 87 consecutive patients that scheduled for surgery under general anesthesia during the study period. After 27 patients were excluded for reasons stated above, 60 patients were randomized to four study groups.

In total, 60 adult patients scheduled for surgery under general anesthesia were enrolled in this study. No patients were excluded from further analysis (Figure 1). Intubation was performed at the first attempt by experienced anesthesiologists in all patients successfully. The demographic data did not differ between the four groups with regard to age and body mass index (BMI). The distribution as per sex, ASA status, hemoglobin, and surgery type was similar in all groups and statistically non-significant. Table 1 shows baseline demographics for each group.

**TABLE 1:** Patient characteristics.

Characteristic	Group LOZP	Group LOHP	Group HOZP	Group HOHP	*p*-Value
Age [years], mean (SD)	51.1 (13.6)	48.6 (12.5)	53.5 (13.6)	54.5 (12.2)	.613
BMI [kg/m^2^], mean (SD)	25.9 (4.0)	26.3 (4.0)	25.7 (3.0)	24.6 (3.8)	.641
Sex, Male (%)	6 (40.0)	11 (73.3)	7 (46.7)	7 (46.7)	.280
ASA (I/II)	1/14	1/14	0/15	0/15	.576
Hb [g/dL], mean (SD)	14.7 (1.6)	15.5 (2.0)	14.9 (2.4)	14.1 (1.9)	.282
History of smoking (Yes/No)	3/12	5/10	5/10	5/10	.824

BMI, body mass index; ASA, american society of anesthesiology, Hb = Hemoglobin.

### 3.1 The non-hypoxemic apnea time is significantly longer in groups applying high FiO_2_ and PEEP

The time from cessation of ventilation to a pulse oximeter reading of 90% (T_100-90_) was 192 s ± 70 s, 221 s ± 74 s, 284 s ± 101 s, and 353 s ± 85 s in the LOZP, LOHP, HOZP, and HOHP group, respectively (F = 10.99, *p* < .01). The T_100-90_ was significantly longer in the HOHP group when compared to other three groups (*p* < .05). And the T_100-90_ of HOZP group was significantly longer than the LOZP group (*p* < .05). The application of 6 cmH_2_O of PEEP did not significantly improve the non-hypoxemic apnea time when compared within the groups receiving the same FiO_2_ (Table 2).

**TABLE 2:** The non-hypoxemic apnea duration and time to return to SpO_2_ of 100%.

Endpoints	Group LOZP	Group LOHP	Group HOZP	Group HOHP	*p*-Value
Non-hypoxemic apnea duration from 100% - 90% [s], mean (SD)	193 (70)	221 (74)	284 (101)	353 (85)	<.01
Non-hypoxemic apnea duration from 100% - 96% [s], mean (SD)	144 (47)	171 (59)	214 (80)	287 (69)	<.01
Non-hypoxemic apnea duration from 96% - 90% [s], mean (SD)	49 (27)	50 (22)	70 (32)	66 (28)	.077
Time to return SpO_2_ of 96% [s], mean (SD)	42 (8)	40 (5)	45 (13)	45 (12)	.444
Time from SpO_2_ of 96% to 100% [s], mean (SD)	19 (16)	16 (12)	45 (54)	29 (21)	.053

**FIGURE 2:**
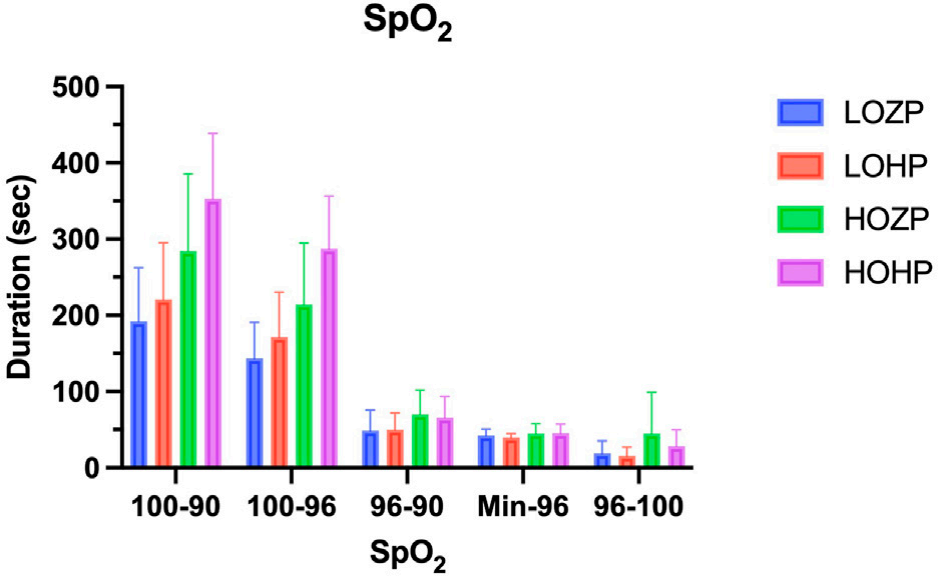
The non-hypoxemic apnea time after induction and the rising time of the SpO_2_ after ventilation resume. The non-hypoxemic apnea time (T_100-90_ and T_100-96_) was significantly longer in the group of HOHP when compared to the other three groups (*p* < .01). The rising time of the SpO_2_ did not differ among the groups (*p* > .05).

The time from cessation of ventilation to a pulse oximeter reading of 96% (T_100-96_) was 144 s ± 47 s, 171 s ± 59 s, 214 s ± 82 s, and 287 s ± 69 s in the LOZP, LOHP, HOZP and HOHP group, respectively (F = 13.90, *p* < .01). T_100-96_ was significantly longer in the HOHP group when compared to the other three groups (*p* < .05). And the T_100-96_ of HOZP group was significantly longer than the LOZP group (*p* < .05). However, the T_100-96_ was not differed between the group of HOZP and LOHP (*p* = .280). The application of 6 cmH_2_O of PEEP significantly improved the T_100-96_ of the group of HOHP when compared to the group of HOZP. The time from a pulse oximeter reading of 96%–90% was slightly longer in groups applying 100% oxygen, but not statistically significant (*p* = .077) (Figure 2).

The lowest pulse oximeter reading after resuming mechanical ventilation did not differ among the four groups (Range from 80 to 89, 25th–75th percentiles 86). The time from resume of mechanical ventilation to a pulse oximeter reading of 96% and 96%–100% did not differ among each group (F = .906, *p* = .444, F = 2.718, *p* = .053, respectively). In group HOZP, the pulse oximeter reading of three subjects took more than 120s to reach 100% from 96%.

### 3.2 PaO_2_/FiO_2_ ratio is better in groups applying low FiO_2_ and PEEP

The PaO_2_ (as well as PaO_2_/FiO_2_ ratio) and PaCO_2_ did not differ among each group prior to ventilation (F = .646, *p* = .589, F = .739, *p* = .533, respectively). The PaO_2_ of the LOZP, LOHP, HOZP, and HOHP group at the time mask ventilation was stopped was 236 ± 71, 298 ± 58, 335 ± 58, 391 ± 50, respectively (F = 16.69, *p* < .01). In the *post hoc* multiple comparisons, the PaO_2_ of the LOZP group was lower than the other three groups (*p* < .05). The PaO_2_ of the HOHP group was significantly higher than groups applying 60% oxygen (*p* < .01). However, the PaO_2_ of the HOZP group did not differ from the LOHP group (*p* = .368), and the PaO_2_ of the HOHP group was slightly higher than the HOZP group, but not statistically significant (*p* = .071). Besides, the results of the PaCO_2_ after ventilation were similar across the groups (F = .185, *p* = .906).

The PaO_2_/FiO_2_ ratio of the LOZP, LOHP, HOZP and HOHP group immediately after ventilation was stopped was 393 ± 130, 496 ± 97, 335 ± 58, 391 ± 50, respectively (F = 8.472, *p* < .001). In the *post hoc* multiple comparisons, the PaO_2_/FiO_2_ ratio of the LOHP group was significantly higher than the HOZP group (*p* = .003), and slightly higher than LOZP group and HOHP group with *p* values of .052 and .079.

**FIGURE 3:**
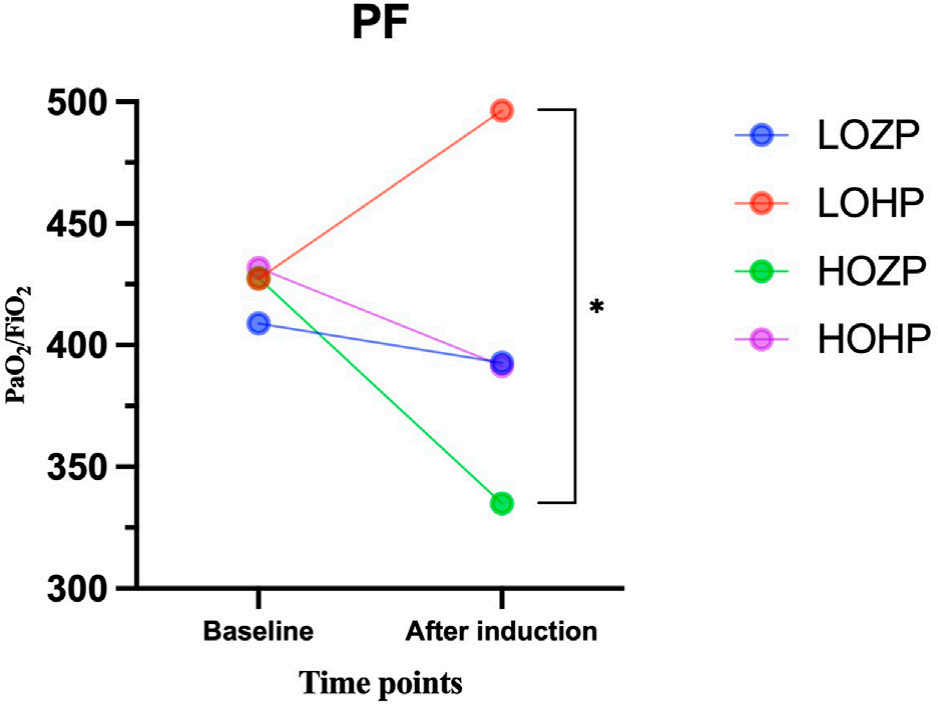
Results of the PaO_2_/FiO_2_ ratio before and after induction of the four groups. The LOHP was significantly improved (*p* < .01), the group LOZP and HOHP remained the same, while the group HOZP significantly decreased (*p* < .01).

When compared the PaO_2_/FiO_2_ ratio after ventilation to its value with the patient breathing room air, the PaO_2_/FiO_2_ ratio in the group of LOHP was significantly improved (*p* < .01), the group LOZP and HOHP remained the same, while the group HOZP significantly decreased (*p* < .01) (Figure 3; Table 3).

**TABLE 3:** The arterial blood gases before and after induction.

Arterial blood gas values	Group LOZP	Group LOHP	Group HOZP	Group HOHP	*p*-Value
Baseline PaO2 [mmHg], mean (SD)	86 (10)	90 (10)	90 (11)	91 (10)	.589
Baseline PaCO2 [mmHg], mean (SD)	40 (2)	39 (3)	38 (4)	38 (3)	.533
Baseline P/F ratio, mean (SD)	409 (47)	427 (47)	428 (54)	432 (48)	.589
PaO2 after induction [mmHg], mean (SD)	236 (78)	298 (58)	335 (58)	391 (50)	<.01
PaCO2 after induction [mmHg], mean (SD)	44 (4)	45 (3)	45 (6)	45 (5)	.906
P/F ratio after induction, mean (SD)	393 (130)	496 (97)	335 (58)	391 (50)	<.01

## 4 Discussion

In this prospective, randomized controlled trial, we evaluated the effects of two levels of PEEP (0 cmH_2_O and 6 cmH_2_O) and two levels of FiO_2_ (1.0 and .6) on non-hypoxemic apnea time and PaO_2_/FiO_2_ ratio during face mask ventilation of anesthesia induction in adults. The major finding of the present study is that, application of 6 cmH_2_O of PEEP and 100% of oxygen during face mask ventilation during general anesthesia induction could maximize the non-hypoxemic apnea time in non-obese, non-critically ill elective surgery patients.

### 4.1 Pure oxygen combined with PEEP could maximize the non-hypoxemic apnea time

An expected finding in our study is that face mask ventilation after induction anesthesia with 100% of oxygen combined with PEEP provided the longest non-hypoxemic apnea time among all four groups. Previous studies have shown that, the use of continuous positive pressure during induction was well-established to decreases atelectasis formation and prolongs the non-hypoxemic apnea time among different populations from infants to adults, the non-obese to obese (Coussa et al., 2004; Gander et al., 2005; Tagaito et al., 2007; Isono, 2009; Kim et al., 2021). The application of PEEP was shown by CT scans to limit the atelectasis formation, therefore provides an increase in functional residual capacity and in the intrapulmonary oxygen store (Barbosa et al., 2014). As shown in our study, ventilation with the use of PEEP and 100% oxygen could more efficiently maintain the duration of non-hypoxic apnea than without PEEP or with a lower FiO_2_. Therefore, in clinical practice, application of PEEP during ventilation should be considered as a standard technique for all anesthesia induction, because it extends the non-hypoxemic apnea time, improves oxygenation, and increases the margin of safety before intubation.

### 4.2 Ventilation with low FiO_2_ and PEEP provide better oxygenation

With the rise of lung protective ventilation strategy in recent years, the needs for better PaO_2_/FiO_2_ ratio during anesthesia induction (or the peri-anesthesia period) are increasing, while anesthesiologists are still seeking better ventilation methods and longer non-hypoxemic apnea time (Young et al., 2019). Therefore, in this study, we tested the application of 6 cmH_2_O of PEEP and FiO_2_ of 60% during mask ventilation of ventilation. We found that the FiO_2_ of 60% and PEEP obtained similar non-hypoxemic apnea time when compared to the classical ventilation methods.

However, the PaO_2_/FiO_2_ ratio after mask ventilation of the two groups above mentioned are totally different. When compared to its value before induction, the LOHP group’s PaO_2_/FiO_2_ ratio increased significantly while the HOZP group decreased significantly. Although the application of 6 cmH_2_O of PEEP and FiO_2_ of 100% obtained the longest non-hypoxemic apnea time in this study, the PaO_2_/FiO_2_ ratio of these patients were not as high as patients receiving 6 cmH_2_O of PEEP and FiO_2_ of 60%. A possible explanation could be that 60% oxygen with PEEP decreases the most amount of atelectasis formation, hence increases the functional residual capacity, and at the same time decreases the level of intrapulmonary shunt. What’s more, there were three subjects in group HOZP took more than 120 s to reach a pulse oximeter reading 100% from 96%. This may also result from massive atelectasis formation especially during apnea period of the study procedure, which lead to a relatively slow recruitment of ventilated alveolus.

### 4.3 Pure oxygen may not always be the best choice during induction

Anesthesia has become significantly safer over the past decades, especially during the establishment of artificial airway (Bainbridge et al., 2012). The application of visualization technology during airway management has made the intubation maneuver much safer (Lewis et al., 2016; Hoshijima et al., 2018). It was reported that the intubation duration was no more than 40 s when performed by experienced anesthesiologists in patients without difficult airway, and almost all the patients could be intubated at the first attempt (Li et al., 2017). Therefore, the classical ventilation methods (100% oxygen and ZEEP) already provide enough non-hypoxemic apnea duration for anesthesiologists to establish artificial airway in most circumstances. In recent years, there has been increasing evidence to suggest that the application of high FiO_2_ during mechanical ventilation can result in a variety of adverse effects (Meyhoff et al., 2012). Studies showed that the administration of pure oxygen after the administration of paralysis cause significantly more atelectasis formation than combined with air (Reber et al., 1996). As the air in the lungs is replaced by 100% oxygen during classical ventilation methods. Marco et al. reported that the atelectasis would develop as soon as the initiation of positive pressure ventilation, to a level of 4.1% ± 2.0% (Lundquist et al., 1995; Coussa et al., 2004). At the same time, the function residual capacity (FRC), which is closely related to the aerated lung volume, would also loss dramatically (Sreejit and Ramkumar, 2015). Moreover, Guniz et al. found that when compared to 40% FiO_2_, 80% FiO_2_ decreased expiratory tidal volumes and PaO_2_/FiO_2_ ratio, increase lactate levels and systematic oxidative stress, inhibiting antioxidant response (Koksal et al., 2016). It is also reported that high FiO_2_ ventilation may increase peripheral vascular and coronary artery vasoconstriction, decrease cardiac output (Harten et al., 2003). However, in patients with signs of difficult airways or any other reasons to require maximal safety during induction, 100% oxygen should always be preferable for anesthesiologists in order to secure the airway and prevent severe hypoxemia.

### 4.4 Risks in this study

First, contradictory to classical techniques, preoxygenation was not carried out in our study to ensure equal oxygenation levels among each group prior to anesthesia induction. Of note, all patients were instructed to take five deep breaths before anesthesia induction to recruit the lungs and optimize the functional residual capacity. Besides, we prefilled the breathing circuit and face mask with oxygen to ensure effective face mask ventilation. Second, to minimize the incident of difficult intubation and ventilation, we eliminated patients with potential risks for difficult airway. The neck and mouth opening were evaluated before any procedures. Complete body examination also was completed before enrollment. During the induction period, in addition to the anesthesiologist in charge, there were also two additional physicians: the anesthesia resident, and the investigator, who is also a senior anesthesiologist. And other intubation tools or ventilation tools such as video stylet, flexible videoscope, and laryngeal mask were also available in the operating room. Third, during 90 s of face mask ventilation, all patients were subjected to two-handed mask ventilation with a “Guedel” style oropharyngeal airway placed. Fourth, a potential risk in our study is that face mask ventilation with PEEP is to expose a paralyzed patient to gastric insufflation thus increase the risk of regurgitation and aspiration.

### 4.5 Limitations

First, we only tested two levels of PEEP (0 cmH_2_O and 6 cmH_2_O) in this study, the best PEEP during ventilation is still unknown. Second, the patients’ EtO_2_ in this study was monitored only visually by the attending anesthesiologist and the resident but it was not recorded during the face mask ventilation. Third, electric impedance tomography (EIT) and computed tomography (CT) were not used to monitor the amount of atelectasis before and after anesthesia induction.

In conclusion, the application of 6 cmH_2_O of PEEP during face mask ventilation of anesthesia induction could extend the non-hypoxemic apnea time, while significantly improving the PaO_2_/FiO_2_ ratio after ventilation when combined with a FiO_2_ of .6.

## Data Availability

The raw data supporting the conclusion of this article will be made available by the authors, without undue reservation.
